# Addressing Teacher Occupational Health in Challenging Times: The Role of a Positive Organizational Climate in Buffering Teachers’ Burnout

**DOI:** 10.3390/ijerph23010042

**Published:** 2025-12-28

**Authors:** Sofia Oliveira, Magda Sofia Roberto, Ana Margarida Veiga-Simão, Alexandra Marques-Pinto

**Affiliations:** 1 Business Research Unit (BRU), ISCTE—Instituto Universitário de Lisboa, 1649-026 Lisbon, Portugal; 2 CICPSI, Faculdade de Psicologia, Universidade de Lisboa, 1649-013 Lisbon, Portugal; msroberto@psicologia.ulisboa.pt (M.S.R.); amsimao@psicologia.ulisboa.pt (A.M.V.-S.); ampinto@psicologia.ulisboa.pt (A.M.-P.)

**Keywords:** burnout, COVID-19, occupational health, organizational climate, teachers

## Abstract

**Highlights:**

**Public health relevance—How does this work relate to a public health issue?**

- Teacher burnout represents a growing public health concern, with implications for teachers’ occupational health, education quality, and student outcomes.
- The study examines how the COVID-19 pandemic intensified pre-existing psychosocial risks in school settings, emphasizing the role of contextual job resources in mitigating teacher burnout.

**Public health significance—Why is this work of significance to public health?**

- By using longitudinal data with a pre-pandemic baseline, this research provides robust evidence on how teacher burnout indicators developed over time, addressing a critical gap in predominantly cross-sectional public health research.
- The findings identify organizational climate and perceived personal accomplishment as key protective factors, informing prevention-oriented approaches to occupational mental health.

**Public health implications—What are the key implications or messages for practitioners, policy makers, and/or researchers in public health?**

- Our results underscore the need to prioritize organizational-level interventions, such as supportive leadership, social support structures, and professional development, to mitigate teacher burnout and promote sustainable working conditions.
- The study highlights the value of longitudinal and context-sensitive designs, supporting the inclusion of positive organizational resources as central components of public health strategies targeting work-related stress.

**Abstract:**

Teacher occupational health is a critical issue worldwide that COVID-19 has worsened. While previous research has highlighted the impact of chronic work-related stress and limited personal resources on burnout, much of this research relies on cross-sectional data that do not capture how these effects develop over time. Additionally, the role of positive organizational factors remains underexplored. Our study examined burnout trajectories among 101 Portuguese elementary teachers (94.1% women, *M* = 46.03 years, 85.6% enrollment rate) over five data collection points spanning the 1st and 2nd COVID-19 waves (2019–2021) and investigated the impact of organizational climate on teacher burnout indicators. Main work-related stressors were identified through an open-ended question. Trajectories of occupational stress and burnout were analyzed using independent ANOVAs, and moderation analyses tested the relationship between organizational climate, occupational stress, and burnout indicators. Results showed a significant drop in perceived personal accomplishment during the first lockdown. Key stressors included greater job demands and more strained interpersonal relationships. Organizational climate significantly moderated the effect of work-related stress on emotional exhaustion, while having a positive main effect on personal accomplishment. This research contributes to a strengthened theoretical understanding of burnout as a dynamic, context-sensitive process, offering new empirical evidence, especially in underrepresented educational systems like Portugal. It emphasizes the importance of addressing contextual factors when working to reduce teacher burnout. Rethinking professional development and workplace relationships is essential for supporting teachers’ occupational health in today’s uncertain educational environments.

## 1. Introduction

Work-related stress is a widely recognized challenge for teachers’ occupational health and job performance [1,2,3], emerging as a primary cause of burnout. In this paper, we follow Maslach’s model, which defines teacher burnout as a three-dimensional syndrome comprising emotional exhaustion (i.e., feeling emotionally depleted by one’s work), depersonalization (i.e., adopting an impersonal and detached attitude toward students), and reduced professional accomplishment (i.e., diminished sense of professional efficacy and fulfillment at work) [4,5]. According to this theoretical perspective, teacher burnout is driven by chronic teaching stressors. These are reflected in the high daily job demands that teachers face, including quantitative and qualitative workload, role conflict, interpersonal conflicts, challenging relationships with colleagues, school leaders, and parents, pupil misbehavior, and unfavorable work schedules and work conditions, while also being pressured to maintain a good job performance and ensure their students’ academic, social, and emotional success [1,6,7]. Building on Lazarus and Folkman’s [8] Transactional Model of Stress and Demerouti et al.’s [9] Job Demands and Resources (JD-R) model, a substantial body of literature highlights how these persistent work-related stressors contribute to teachers’ chronic occupational stress and ultimately to burnout (e.g., [3,5,7,10,11,12,13]).

Teachers’ occupational health is thus a critical global issue today. A systematic review by von der Embse et al. [14] reported that approximately 30% of teachers experienced clinically significant levels of stress, a number that has increased drastically in recent years [15]. With teachers experiencing above-average levels of occupational stress for decades [16], many educational systems are struggling to retain qualified teachers. Chronic work-related stress and burnout lead to negative affect, reduced job satisfaction, diminished teaching effectiveness, and higher attrition rates and turnover [17,18]. These factors are fueling a growing teacher retention crisis worldwide, which has become a central policy concern with significant repercussions for education quality and student outcomes [19,20]. Addressing these interconnected problems demands a clear understanding, both theoretically and empirically, of the mechanisms underlying teacher work-related stress and burnout, including the individual, contextual, and temporal factors that may shape them. There is, therefore, at both a political and social level, a pressing need to address teachers’ occupational health to enhance attractiveness and improve retention. Teachers’ demographic characteristics also warrant attention as prior studies indicate that stress levels vary by educational level taught, gender, and career stage, with younger and female teachers in elementary education often reporting greater vulnerability (e.g., [21]).

Moreover, teachers’ occupational stress does not occur in a vacuum, but it is significantly amplified by macro-level societal disruptions, such as the COVID-19 pandemic [15,22]. Although teachers have long been performing roles beyond “traditional” teaching (e.g., [23,24]), these demands grew during the pandemic [25], increasing teachers’ occupational health risks [26,27]. In today’s increasingly BANI (Brittle, Anxious, Nonlinear, and Incomprehensible) world, marked by instability and ambiguity, it is essential to understand how teachers adapt to and are impacted by ongoing uncertain and structural changes. This understanding is vital for preparing educational systems to better support teachers during challenging times. To do so, we need not only to understand teachers’ job demands during times of adversity, but also to learn how to better empower teachers to handle these strains. Furthermore, despite the growing recognition that teacher burnout varies across sociocultural settings (e.g., [28,29]) and the consistent reports that Portuguese employees are particularly vulnerable to burnout [30], especially in the education sector [31,32,33,34], evidence from the Portuguese educational contexts remains scarce (e.g., [35]). Available data indicate that Portuguese teachers experience above-average stress levels and significant health risks, with long working hours, low salaries, heavy administrative workload, and job dissatisfaction being cited as key psychosocial risk factors [21].

Accordingly, our study aimed to investigate the evolution of Portuguese elementary school teachers’ occupational health between Fall 2019 and Summer 2021. Additionally, we explored the supportive role of contextual job resources, namely organizational climate, in buffering the impact of occupational stress on burnout indicators during this period. In this paper, we define organizational climate as teachers’ shared perception of the unique characteristics that compose their work environment, as a result of their experience of organizational norms, goals, values, interpersonal relationships, procedures, and practices [36].

Although our data captures a historically bounded context during the peak and immediate aftermath of the COVID-19 pandemic, the structural and psychological pressures experienced by teachers have not ended; instead, they have persisted and evolved into a “new normal” marked by heightened demands, digitalization, and intensified accountability (e.g., [31]). Recent global reports further indicate that pandemic-induced work-related stressors have persisted beyond the crisis peak, becoming embedded in teachers’ everyday realities [19,37]. With the pandemic catalyzing disruptions in teaching, teachers’ professional reality is now, more than ever, shaped by a fast-paced and shifting context [37]. Thus, while temporally anchored in the pandemic era, our findings remain relevant for informing strategic policies and practices aimed at supporting teacher occupational health within the increasingly volatile and demanding post-pandemic educational systems.

### 1.1. Impacts of COVID-19 on Teacher Occupational Health

Pandemics have historically disrupted social systems, and COVID-19 was no exception, posing the most significant global challenge in a generation. As a result, researchers worldwide have studied its effects on health, economy, and daily life [38]. Among its many impacts, it profoundly disrupted education systems, placing unprecedented demands on teachers worldwide [37]. The sudden shift to emergency remote teaching required educators to rapidly adapt to new instructional methods while managing the emotional toll of working in a crisis [39,40,41]. The pandemic has intensified job demands, diminished professional efficacy, and placed additional strain on teacher-student and teacher-colleague interactions, all of which are critical factors in occupational health and job satisfaction [22,37]. Specifically, prior studies have described the strain experienced by teachers due to increased and emerging job demands, such as transitioning to online learning and technological constraints [25,39,40,41,42,43], an increased workload [41,42,43,44], difficulties with communication and interpersonal relationships with students and their families [25,42,45], and work–life conflict [15,40,41]. These studies also reported high levels of teacher stress and burnout [15,26,41,44,46,47], and a decrease in teachers’ occupational well-being [43], enthusiasm [46], and sense of success [40] during the pandemic. A growing body of research also suggests that the decline in teachers’ sense of professional success during the pandemic is often accompanied by heightened stress, frustration, and emotional exhaustion (e.g., [40,48]).

Drawing on the Social Cognitive Theory [49], teachers’ reduced personal accomplishment (i.e., also referred to as low sense of professional efficacy) plays a central role in the burnout process [50]. Persistent exposure to work-related stressors can erode teachers’ confidence in their ability to meet job demands, triggering burnout. While no single developmental model of burnout is universally accepted, diminished professional efficacy is a consistent and inevitable factor [51]. Recent research further identifies distinct burnout profiles, illustrating how enthusiasm, commitment, and engagement may progressively deteriorate over time, leading to a reduced sense of professional efficacy and reliance on passive coping strategies [52,53]. Understanding how these processes unfolded during the pandemic offers valuable insights for decision-making aimed at strengthening teachers’ personal accomplishment and engagement, ultimately helping prevent burnout in future crises. However, most of these empirical studies employed cross-sectional designs, capturing isolated snapshots of a complex and evolving phenomenon (e.g., fatigue levels during lockdowns) [41,45,54]. Burnout, particularly emotional exhaustion and reduced personal accomplishment indicators, is conceptualized as a dynamic, time-sensitive phenomenon that fluctuates in response to evolving job demands and resources [5]. Cross-sectional data are therefore insufficient to capture whether burnout experiences intensify, plateau, or fade over time, or how contextual factors like organizational climate affect various stages of burnout development. Therefore, longitudinal designs (e.g., [42,46]) are necessary not only to model burnout development patterns across critical transitions but also to test whether protective factors such as a positive organizational climate may impact this trajectory.

**Figure 1:**
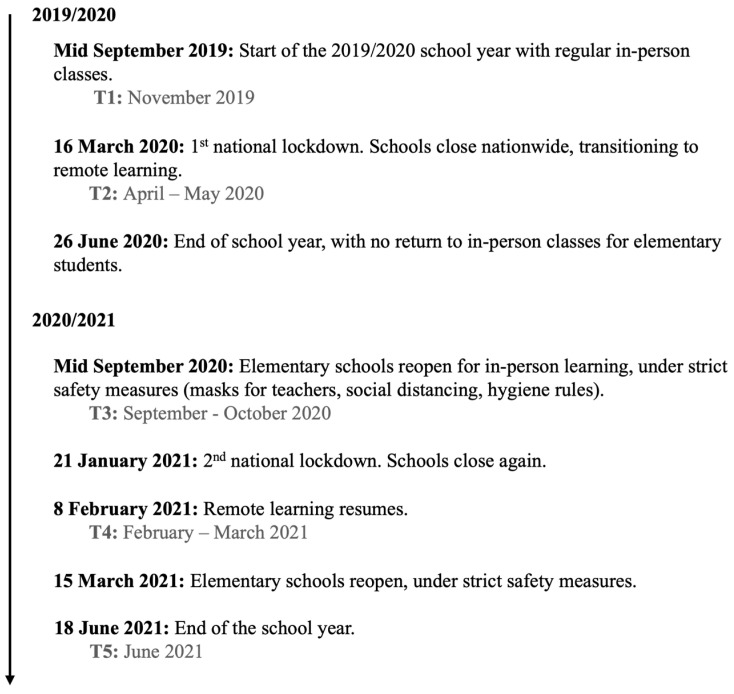
Timeline of key events during 2019/2020 and 2020/2021 school years, and research data collection points.

Furthermore, despite growing recognition that teacher burnout varies across sociocultural settings (e.g., [28,29]), most empirical work has been conducted in Anglo-American or Northern European educational systems. These findings may not generalize to Southern European contexts like Portugal, where the educational system is characterized by a distinct organizational structure and socio-political context [55]. During the 2019/2020 and 2020/2021 school years, Portuguese elementary schools were affected by several key COVID-19 events, including a sequence of closures, partial reopenings, and stringent health protocols (see Figure 1). Although the pandemic affected all educational levels, national studies have shown that the occupational health of elementary school teachers was most significantly impacted [43], likely due to the intensive caregiving and affective labor involved in early education [27]. During these shifting conditions, teachers had to adapt and continue to teach while facing increased job demands and personal and social challenges. Whilst navigating the two national lockdowns, Portuguese teachers faced unique stressors trying to maintain educational quality and support students’ well-being. Moreover, the Portuguese educational system faces unique organizational challenges, including high workloads, centralized governance, and an aging workforce, which may interact and shape work-related stress and burnout differently [24,31]. Addressing these context-specific dynamics is not only important for local policy and intervention efforts but also broadens the cross-cultural validity of occupational health models in education [28].

### 1.2. The Role of Contextual Job Resources

Anchored in the JD-R model, burnout stems from chronic experiences of work-related stress, caused by a perceived prolonged and cumulative imbalance between excessive job demands and insufficient job and personal resources available to manage them [1,6]. The JD-R model identifies two distinct processes through which job demands and resources influence employees’ occupational health: (1) the health impairment process in which excessive job demands, when not properly managed, lead to strain and exhaustion, and (2) the motivational process in which the presence of high job and personal resources promotes increased motivation, work engagement, and performance [6,56]. Even though the three burnout dimensions (i.e., emotional exhaustion, depersonalization, and personal accomplishment) are interrelated, they may emerge differently from these processes. Emotional exhaustion and depersonalization appear to primarily reflect the health impairment pathway and are most sensitive to job and personal resources that moderate the strain process, helping individuals to manage job demands [6]. In contrast, personal accomplishment appears to reflect the motivational process to a greater extent and is therefore most strongly related to job resources that enhance competence, recognition, and growth opportunities [6,41]. Moreover, while job demands are often structurally embedded and difficult to eliminate, enhancing personal and job resources represents a viable and evidence-based strategy for sustaining teachers’ occupational health, helping them cope with job demands, and maintaining motivation.

Research consistently shows that workplace conditions have a significant impact on teachers’ occupational health [7]. Social aspects of the work environment have a particularly strong influence on teachers’ occupational health [57]. Specifically, social support, leadership quality, collegial relationships, and a positive organizational climate have all been shown to promote teacher occupational health and well-being [58,59]. In highly relational professional environments such as teaching, exchanges in social interactions with students, colleagues, superiors, parents, and school staff are frequent, making positive social relations essential for fulfilling teachers’ need for social relatedness [60]. Positive relationships with colleagues and parents appear to have a particularly significant impact on teachers’ work experiences [57].

In this scenario, organizational climate (i.e., teachers’ shared perception of the unique characteristics that compose their work environment [36]) has emerged as a critical job resource. Empirical studies link positive organizational climate to higher job satisfaction, efficacy, and occupational health [12,61]. During the pandemic, studies reinforced the significance of social workplace aspects in sustaining teachers’ well-being [57,62]. Specifically, supportive work environments and social support from colleagues and leaders were consistently identified as important job resources during this period [39,40,42,44,45]. Blaydes et al. [42] found that helpful actions from school leaders, colleagues, and parents were the teachers’ most reported resources during COVID-19. This evidence suggests that a positive organizational climate can potentially buffer teacher burnout by mitigating emotional exhaustion, reducing depersonalization, and enhancing personal accomplishment in high-stress environments. However, most of this evidence is based on short-term or cross-sectional designs and qualitative data, leaving unanswered questions about how organizational climate functions as a sustained protective factor over extended periods of disruption. This question is particularly pressing in educational systems where social and institutional support mechanisms are already constrained. In Portuguese elementary schools, where, alongside high levels of teacher burnout [31,32,33,34], teachers report limited access to collaborative professional cultures and supportive leadership [24,36,63], the buffering potential of a positive organizational climate may be even more critical.

### 1.3. The Present Study

While many studies have documented the immediate impacts of the pandemic on teachers’ outcomes, few have explored how teacher work-related stress and occupational health evolved throughout prolonged disruptions [18]. Likewise, there remains a scarcity of research addressing how teachers’ occupational stress and health fluctuate throughout the school year [18]. Understanding the long-term impact of major disruptions on occupational health is essential, particularly in today’s BANI world. Policymakers and schools must proactively strengthen teachers’ job and personal resources not only to react to disruption but to maintain teachers’ occupational health and well-being. One key protective factor is organizational climate, an essential job resource that encompasses leadership practices, colleagues’ and supervisors’ social support, and workplace policies that influence teacher well-being and performance [61]. Previous research has demonstrated that a positive organizational climate can buffer occupational stress and burnout and foster teachers’ personal resources [63]. However, its role in mitigating work-related stress and burnout during extended periods of disruption remains insufficiently explored.

This study addresses these gaps by examining the occupational health trajectories of Portuguese elementary school teachers across two pandemic-affected school years. By leveraging a multi-wave design, we were able to capture both immediate and extended impacts of COVID-19, going beyond the common static snapshots in post-pandemic educational research. Our study also contributes to existing literature by explicitly examining the protective role of job resources during ongoing disruptions and changes, which remains an understudied topic [22,64]. Building on previous literature, we explored the role of contextual job resources, namely organizational climate, in supporting teachers’ occupational health, particularly during this period. Thus, our study addressed the following research questions:

**Q1.**  *How did teachers’ occupational stress and burnout indicators vary throughout two school years during the COVID-19 pandemic?*

**Q2.**  *What were the main work-related stressors experienced by teachers during this period?*

**Q3.**  *How did the social environment of the workplace impact teachers’ occupational health during the pandemic?*

Building on these research questions, we argue that organizational climate interacts with occupational stress to explain teacher burnout indicators. Thus, the following hypotheses are postulated:

**Hypothesis 1a (H1a).** 
*Organizational climate moderates the relationship between occupational stress and emotional exhaustion, such that this relationship is weaker under high positive organizational climate compared to low.*

**Hypothesis 1b (H1b).** 
*Organizational climate moderates the relationship between occupational stress and depersonalization, such that this relationship is weaker under high positive organizational climate compared to low.*

**Hypothesis 1c (H1c).** 
*Organizational climate moderates the relationship between occupational stress and personal accomplishment, such that the negative impact of work-related stress on personal accomplishment is weaker under high positive organizational climate compared to low.*

By answering these questions, our study contributes to a more nuanced and longitudinal understanding of how work-related stressors and organizational climate shape teacher burnout trajectories in times of prolonged disruption, providing insights into strategies for supporting educators during challenging times. The findings aim to inform future policies and interventions aimed at fostering healthier and more sustainable work environments for teachers, ultimately enhancing both educators’ retention and performance, as well as students’ academic success.

## 2. Method

### 2.1. Participants and Procedure

A convenience sample of 101 elementary school Portuguese teachers (94.1% women, *M* = 46.03 years, *SD* = 5.12, range: 28–62 years) participated in the study. Participants were practicing at 14 state elementary schools across three different school clusters in the Lisbon Metropolitan Area. Teachers had a mean of 15.69 years of overall teaching experience (*SD* = 8.56, range: 1–36), with an average of 8.95 years (*SD* = 6.92, range: 1–24) at their current school cluster. Most teachers (98.48%) had permanent contracts and held a degree (93.9%). The three school clusters had similar organizational structures, socioeconomic level, and size. The total eligible population of the school clusters was 118 teachers, and after ensuring voluntary participation, the enrollment rate was 85.6%.

We collected data through an online survey using the Qualtrics platform. As the pandemic was unforeseen, stress and burnout trajectories were assessed using a five-wave repeated cross-sectional design: T1 (pre-pandemic, Nov 2019, *n* = 66), T2 (1st lockdown, Apr/May 2020, *n* = 73), T3 (return to in-person teaching, Sep/Oct 2020, *n* = 81), T4 (2nd lockdown, Feb/Mar 2021, *n* = 76), and T5 (end of school year back to in-person teaching, Jun 2021, *n* = 74). Across all waves, the study included a total of 101 unique teachers. This cumulative sample consisted of the 66 teachers recruited at baseline (T1) and an additional 35 teachers who enrolled in the study at later data collection points. Thus, the sample sizes at individual waves are smaller than the overall total, reflecting both staggered entry into the study and attrition over time. To mitigate the limitations of independent samples and increase transparency regarding sample overlap, we asked participants at T2 and T3 whether they had taken part in previous data collection waves. Of the 66 teachers initially enrolled at T1, 65 also participated in T2 (98.5%), and 57 continued to participate from T3 onwards (86.4%). Teachers who joined the study after T1 contributed data exclusively to subsequent waves. For moderation analysis, we only included participants with paired data across three consecutive waves (T3–T5), yielding a longitudinal subsample nested within the broader repeated cross-sectional framework. Data from T3 onwards were paired using an alphanumeric code.

Teachers were contacted via email, informed of the study’s purpose, and asked for their consent to participate. Voluntary participation and the possibility of dropout were guaranteed, and confidentiality and anonymity of the data were ensured. The survey lasted approximately 15 min, and validity checks were included to ensure the accuracy of responses. Only complete responses were considered. Text entry boxes were used to collect sociodemographic data, which helped detect random responses, spam, or the use of autofill software. Additionally, a statement encouraging honesty was included to reduce social desirability bias. The authors had no prior relationship with the participants, and no compensation was offered.

### 2.2. Measures

#### 2.2.1. Perceived Occupational Stress

Overall occupational stress was measured, at all data collection waves, with a single item (adapted from Kyriacou & Sutcliffe [2]) by asking teachers, “To what extent do you consider that being a teacher, in general, was a stress-generating activity over the past two months?” The item was rated on a 5-point scale (1—*Not stress-generating at all* to 5—*Extremely stress-generating*). At T2, teachers also identified up to 10 main workplace stressors experienced during the first COVID-19 lockdown (in an open-ended question), rating the frequency of each on a 7-point scale (0—*Never* to 6—*Every day*).

#### 2.2.2. Burnout

We used the Portuguese adaptation of the Maslach Burnout Inventory—Educators Survey [65] to measure burnout indicators, at all data collection waves, through 22 items (e.g., “I feel emotionally drained by my work.”) addressing Emotional exhaustion (0.85 < *ω*_T1–T5_ < 0.93), Depersonalization (0.68 < *ω*_T1–T5_ < 0.88), and Personal accomplishment (0.65 < *ω*_T1–T5_ < 0.84). Answers were given on a 7-point Likert scale (0—*Never* to 6—*Every day*).

#### 2.2.3. Organizational Climate

Teachers’ perceptions of their school climate were assessed using the 40-item Portuguese version of the Organizational Climate Description Questionnaire Revised for Elementary Schools (0.86 < *ω*_T2–T5_ < 0.88) [36]. Items (e.g., “Teachers help and support each other.”) assessed the quality of teacher-teacher (i.e., collegial, intimate, and/or disengaged) and teacher-principal interactions (i.e., supportive, directive, and/or restrictive) [36] and were evaluated on a 4-point scale (1—*Rarely occurs* to 4—*Very frequently occurs*). Organizational climate was only measured at T2 and subsequently.

### 2.3. Data Analysis

We conducted a mixed deductive/inductive thematic content analysis using MAXQDA 24 software to analyze perceived workplace stressors following Bardin’s guidelines [66] and the JD-R model [7,9]. Deductive analysis identified initial categories (e.g., quantitative and qualitative workload), while inductive analysis of specific demands and (lack of) resources emerging from teachers’ responses further refined and consolidated the category system (e.g., challenging parent-teacher interactions). To ensure the validity of the analysis, the assumptions of exclusivity, homogeneity, pertinence, objectivity, and productivity were assured during the coding process [66]. To ensure reliability, an independent coder reviewed the category system and discussed divergences with the first author until full agreement was attained. We used MAXQDA’s Code Relations Browser to explore relationships within the (sub-)category system [67]. We observed sub-category co-occurrences for each participant to facilitate the identification of patterns, interactions, and interpretation of the data [68]. A frequency analysis of teachers’ perceived stressors was conducted using SPSS Statistics 29.

For hypothesis testing, we used *R* (version 4.4.1) [69]. Descriptive statistics and preliminary analyses were employed to examine data distribution, identify missing values, and detect outliers. As the software notified participants of the need to complete their responses before submission, there were no missing values. *Q-Q* plot analysis indicated a near-normal distribution of the data (i.e., |z| < 3) [70]. Internal consistency was assessed with coefficient omega (*ω*), with values ≥ 0.70 considered good [71]. Owing to the sample size within the data collection points, all analyses were performed using robust statistics. Robust one-way ANOVAs based on trimmed means (20% trimming) and post hoc tests (*WRS2* package; [72]) were performed to assess changes in occupational stress and burnout indicators over time (T1–T5). To account for family-wise error rates, the Holm-Bonferroni method was used for multiple comparisons [72,73]. To examine the relationship between organizational climate and burnout indicators, Spearman correlations were computed, and robust linear regression models were performed (*robustlmm* package; [74]) to evaluate whether perceived organizational climate explained the variance of teachers’ burnout indicators at T2. Values around 0.10, 0.30, and 0.50 illustrate small, moderate, and large associations, respectively [75].

Robust multiple regression, conducted with the *robustbase* package for R [73], was employed to explore the moderating effect of organizational climate measured at T4 on the relationship between occupational stress measured at T3 and burnout indicators measured at T5. The interaction term between occupational stress and organizational climate was included. Variables were mean-centered to reduce multicollinearity. Simple slope analyses were conducted to interpret the nature of moderation at different levels of the moderator. Effect sizes with 95% CI with bootstrap were calculated for robust one-way ANOVAs using the Partial Eta Squared (*η*^2^), and for robust regression using Cohen’s *f* (*f*^2^). All analyses were conducted with a significance level of *p* < 0.05 whenever the 95% bootstrapped CI did not include 0.

## 3. Results

### 3.1. Occupational Stress and Burnout Indicators Evolution Across Time: Five-Wave Repeated Cross-Sectional Study

We found statistically significant differences for personal accomplishment (*F*(4, 109.57) = 3.41, *p* = 0.011, *η*^2^ = 0.28, 95% CI [0.16, 0.47]), with teachers reporting a significant decrease in sense of professional efficacy in the first lockdown, compared with the other four data collection points (T1: $\hat{\Psi }$ = −0.46, 95% CI [−0.90, −0.02], T3: $\hat{\Psi }$ = 0.48, 95% CI [0.06, 0.90], T4: $\hat{\Psi }$ = 0.49, 95% CI [0.05, 0.92], T5: $\hat{\Psi }$ = 0.46, 95% CI [0.02, 0.90]). Despite slight variations in the mean scores (Table 1) for the remaining variables, no statistically significant differences were found regarding occupational stress (*F*(4, 108.15) = 0.75, *p* = 0.563, *η*^2^ = 0.15, 95% CI [0.06, 0.25]), emotional exhaustion (*F*(4, 109.83) = 0.63, *p* = 0.645, *η*^2^ = 0.16, 95% CI [0.06, 0.26]), and depersonalization (*F*(4, 105.97) = 1.13, *p* = 0.346, *η*^2^ = 0.17, 95% CI [0.08, 0.29]) across the assessed period.

### 3.2. Main Workplace Stressors Experienced During COVID-19

Analysis produced 18 sub-categories of main workplace stressors experienced during COVID-19 (13 sub-categories referred to job demands, one category related to broader social demands, and four sub-categories depicted negative outcomes), with a total of 293 recording units coded (see Supplemental Table S1 for details). Among job demands, teachers perceived Quantitative Workload (70.9%) and Demanding Interactions with students (45.5%) and their parents (41.8%) as the most prominent sources of strain. These stressors frequently co-occurred with Work Reorganization (43.6%) and Unfavorable Work Conditions (25.5%), suggesting that the demands for constant structural and procedural adaptations increased the burden on teachers. Furthermore, IT constraints (38.2%) associated with the use of technology for teaching both by teachers and their students were also frequently referred to by teachers, highlighting the digital challenges in teaching environments. Work–life conflict (16.4%) was also reported, and it co-occurred with teachers’ references to Qualitative Work Overload (21.8%), Emotional Demands at work (12.7%), and Unfavorable Work Schedules (18.2%).

On the other hand, the (Lack of) Social support by colleagues and superiors, with feelings of work isolation (16.4%), was the only job resource to be mentioned by the teachers as a frequent source of stress. References to (Lack of) Social support co-occurred with various job demands (e.g., quantitative workload, demanding interactions and interpersonal conflicts, work reorganization, inadequate work conditions). Along with these direct workplace stressors, teachers also reported two main negative outcomes as causing increased strain: Physical and mental health complaints (12.7%) and Negative emotions (10.9%).

Teachers reported encountering most of the 18 identified stressors weekly, with a median frequency of 4.00. Regarding demographic characteristics, male teachers reported mainly Quantitative workload, and did not refer to strain related to Qualitative work overload, Unfavorable Work Schedules, Emotional demands, or Interpersonal conflicts. In addition, younger teachers reported more Work–life conflict and Unfavorable Work Schedules. In comparison, older teachers reported more Demanding Interactions with parents and strain related to Work Reorganization, Qualitative work overload, and IT constraints.

### 3.3. Relation Between Organizational Climate, Work-Related Stress, and Burnout Indicators: Three-Wave Longitudinal Study

Correlation analysis (Table 2) revealed a positive, moderate, and significant association between organizational climate and personal accomplishment at T2. Subsequent robust linear regression analysis (at T2) indicated that organizational climate explained 13% of the variance in personal accomplishment (*B* = 0.74, *SE* = 0.32, 95% CI [0.09, 1.38], *p* = 0.025, *R*^2^ = 0.13, *f*^2^ = 0.14) but did not account for significant variance in emotional exhaustion or depersonalization. Occupational stress explained 28% of the variance in emotional exhaustion (*B* = 0.70, *SE* = 0.12, 95% CI [0.46, 0.94], *p* < 0.001, *R*^2^ = 0.28, *f*^2^ = 0.39). Emotional exhaustion explained 10% of the variance in depersonalization (*B* = 0.24, *SE* = 0.09, 95% CI [0.05, 0.42], *p* = 0.015, *R*^2^ = 0.10, *f*^2^ = 0.11), whereas occupational stress did not account for a significant proportion of variance in depersonalization. Emotional exhaustion and depersonalization did not account for significant variance in personal accomplishment.

#### Moderation Analysis

Following a longitudinal design, the following moderation analyses tested whether organizational climate at T4 moderated the effect of occupational stress at T3 on burnout indicators at T5.

(1) Emotional Exhaustion

**Figure 2:**
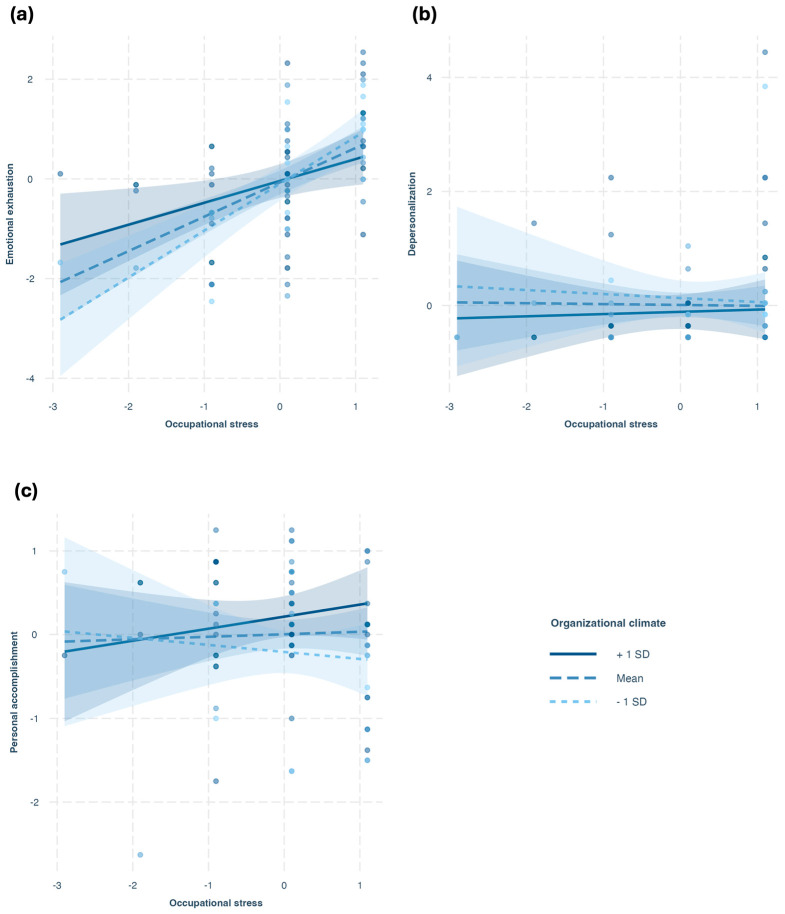
Graphical representation of the simple slopes analyses illustrating the interaction effect of organizational climate (at T4) and occupational stress (at T3) on burnout indicators (at T5). The subgraphs represent the three burnout indicators: (**a**) emotional exhaustion, (**b**) depersonalization, and (**c**) personal accomplishment.

The interaction between occupational stress and organizational climate was significant (*B* = −0.83, *SE* = 0.35, *t* = −2.41, *p* = 0.018), indicating that the effect of occupational stress on emotional exhaustion depended on organizational climate (Figure 2a). There was a significant main effect of stress (*B* = 0.68, *SE* = 0.15, *t* = 4.51, *p* < 0.001). In contrast, the main effect of organizational climate was not significant (*B* = 0.07, *SE* = 0.36, *t* = 0.19, *p* = 0.85). The model explained 30% of the variance in emotional exhaustion [*F*(3, 71) = 11.66, *p* < 0.001, adjusted *R*^2^ = 0.30].

Analysis of simple slopes showed that the positive relationship between occupational stress and emotional exhaustion was strongest at low levels of organizational climate (*B* = 0.94, *SE* = 0.17, *t* = 5.40, *p* < 0.001), and weakest at a more positive organizational climate (*B* = 0.44, *SE* = 0.18, *t* = 2.50, *p* = 0.01). This pattern indicates that as the organizational climate becomes more positive, the impact of work-related stress on emotional exhaustion becomes weaker.

(2) Depersonalization

For this model, following prior literature (e.g., [51]), we tested two predictors of depersonalization, namely occupational stress and emotional exhaustion. The interactions between organizational climate and occupational stress (*p* = 0.712) and emotional exhaustion (*p* = 0.936) were not significant, indicating that the effects on depersonalization did not depend on organizational climate (Figure 2b). There was a significant main effect of emotional exhaustion (*B* = 0.30, *SE* = 0.10, *t* = 2.87, *p* = 0.005) on depersonalization, but not of occupational stress or organizational climate. The model explained 10% of the variance in depersonalization [*F*(5, 70) = 2.67, *p* = 0.028, adjusted *R*^2^ = 0.10].

(3) Personal Accomplishment

Following prior literature (e.g., [51]), we tested whether occupational stress, emotional exhaustion, and depersonalization predicted personal accomplishment, and whether organizational climate moderated these relationships. The overall model was significant, explaining 15% of the variance in personal accomplishment [*F*(7, 68) = 2.94, *p* = 0.010, adjusted *R*^2^ = 0.15], with significant main effects of organizational climate (*B* = 0.70, *SE* = 0.29, *t* = 2.37, *p* = 0.021) and depersonalization (*B* = −0.31, *SE* = 0.10, *t* = −3.01, *p* = 0.004) on personal accomplishment (Figure 2c). However, none of the interaction terms with organizational climate (i.e., moderation effects) reached significance (*p* > 0.05), indicating no evidence that organizational climate moderated the impact of work-related stress, emotional exhaustion, or depersonalization on personal accomplishment.

## 4. Discussion

Our study aimed to understand how Portuguese elementary-school teachers’ occupational health, specifically their occupational stress and burnout indicators, evolved throughout the COVID-19 pandemic (from Fall 2019 to Summer 2021), and to investigate how the social environment in the workplace, particularly organizational climate, related to these outcomes over time. Grounded in the JD-R model and the Social Cognitive Theory, this study addressed three research questions and tested three hypotheses regarding the moderating effect of positive organizational climate on the relationship between occupational stress and burnout indicators. Below, we discuss our findings in relation to each question and highlight their theoretical and practical implications.

**Q1.**  *How Did Teachers’ Occupational Stress and Burnout Indicators Vary Throughout Two School Years During the COVID-19 Pandemic?*

Our results revealed a significant decrease in teachers’ sense of personal accomplishment during the first lockdown (Spring 2020), compared to the pre-pandemic semester (Fall 2019) and the subsequent periods (Fall 2020, Spring 2021, and Summer 2021). The observed pattern is consistent with evidence suggesting that the perception of high job demands (which peaked at this time of data collection) and insufficient job and personal resources will primarily initiate a teacher’s professional efficacy crisis [50]. These findings align with previous research on teacher burnout during the pandemic, which suggests a decline in teachers’ sense of success and enthusiasm [40,46]. Over time, not feeling competent to perform their teaching duties may negatively affect teacher distress, potentially setting in motion a broader process of burnout escalation [50,52]. While our study does not examine this specific predictive analysis, our results are consistent with recent findings indicating increasing levels of teacher burnout after the pandemic’s most acute phases [19,32]. Similarly, a recent post-pandemic survey found that teachers with high efficacy exhibited lower burnout indicators [48]. These results underscore the need to reinforce teachers’ personal accomplishment as a protective factor during times of disruption. To better support teachers in the new, unforeseen, and increasingly demanding teachers’ professional realities, policymakers, school leaders, and practitioners should act to equip teachers with personal and job resources that promote teachers’ perceived professional efficacy [37]. In this regard, our qualitative findings on the main workplace stressors experienced during COVID-19 helped draw on the main challenges and resources to act upon.

In contrast, and contradicting prior literature (e.g., [44,46,47]), no statistically significant differences were observed for emotional exhaustion and depersonalization across the assessed periods. Nevertheless, a slight increase in emotional exhaustion was observed during the first lockdown, surpassing the midpoint of the scale (*M* = 3.03) at T2. This may indicate an initial response to heightened demands that had not yet fully manifested, given that exhaustion is typically associated with prolonged exposure to chronic stress [4,50,52]. The absence of change in depersonalization was less surprising as previous studies have also reported lower levels and smaller effects for this dimension (e.g., [41,47]). This is consistent with theoretical models of burnout development, which suggest that depersonalization is a coping strategy that emerges later, in response to sustained exhaustion rather than acute disruption [4,52]. Possible influential methodological constraints should also be acknowledged. To answer this research question, we used five-wave repeated cross-sectional data, which may be less sensitive to within-person change over time. Additionally, socially desirable response bias, which is particularly prevalent among professional groups characterized by strong norms of dedication and resilience, as well as the possibility that emotional exhaustion and depersonalization were already structurally embedded within teachers’ work contexts, may have obscured short-term fluctuations in relation to efficacy-related perceptions. In sum, the sudden and acute disruption associated with the first lockdown may have been sufficient to undermine teachers’ sense of professional accomplishment, while not yet allowing enough time for exhaustion or cynical distancing to consolidate.

**Q2.**  *What Were the Main Work-related Stressors Experienced by Teachers During This Period?*

Consistent with international evidence, our findings indicated that Portuguese elementary school teachers experienced a wide array of work-related challenges during the COVID-19 outbreak. Thematic content analysis revealed that elementary school teachers experienced significant strain arising from quantitative and qualitative workload, demanding interactions with students and parents, work reorganization, unfavorable work conditions and schedules, IT constraints, work–life conflict, and emotional demands. These findings are consistent with prior literature (e.g., [15,25,39,40,41,42,43,44,45]). Notably, most of these demands (e.g., emotional demands, negative work-to-life spillover, challenges in work–life segmentation, excessive workload) mirror long-standing, well-documented stressors within the teaching profession (e.g., [1,3,7]), suggesting that the pandemic functioned not as a generator of new stressors, but as an amplifier of pre-existing structural vulnerabilities. This distinction is critical, as it underscores the systemic nature of teacher occupational stress and suggests that mitigation strategies should extend beyond temporary pandemic-related measures.

Moreover, several of the most reported stressors (e.g., workload) have been consistently associated with impaired occupational health and well-being, lower job satisfaction, and increased turnover intentions among teachers [12,16]. While their salience may have peaked during the lockdown, these work-related stressors appear to transcend the remote work context. They should therefore be addressed within broader, long-term occupational health frameworks for the education sector.

Nevertheless, the transition to home-office and remote work experienced in T2, as well as the unprepared transition to distance learning, may have exacerbated and helped explain certain challenges, particularly those linked to IT constraints, Work reorganization, and Work–life conflict. These findings align with other literature on the impacts of the pandemic, with teachers worldwide experiencing discomfort and unreadiness for accessing and using the necessary technological tools to teach remotely [37], which also impacted their ability to respond to professional responsibilities [40]. Our findings suggest that difficulties with digital tools, platforms, and connectivity were more frequent among older teachers, aligning with prior evidence on generational divides in digital literacy [40]. Moreover, younger and mid-career teachers reported more difficulties in reconciling work with other personal life responsibilities, suggesting that this demographic may be particularly burdened by competing professional and domestic roles during the pandemic [40].

The (Lack of) Social support stood out as the only job resource cited recurrently as a source of teacher distress. Teachers described feeling isolated at work and missing social support from their colleagues and superiors. Importantly, references to (Lack of) Social support co-occurred with reports on the most frequent job demands previously depicted (e.g., workload, demanding interactions, work reorganization, inadequate work conditions), suggesting a compounding effect in which the absence of relational support might exacerbate the detrimental impact of job demands. These findings reinforce the importance of relational and contextual job resources, such as positive leadership practices, colleagues’ and supervisors’ social support, and adequate workplace policies (i.e., a positive organizational climate) [61], in mitigating teacher occupational stress and burnout. Conversely, the lack of these job resources may intensify the negative impact of job demands on teacher occupational health. These findings are consistent with evidence indicating that supportive school leadership plays an important role in offsetting the adverse effects of workplace pressure and work overload [76] and that positive relationships with colleagues are associated with job satisfaction and decreased turnover intention [77]. They also corroborate prior research, which identified these factors as important job resources for sustaining teacher occupational health during the pandemic [39,40,42,44,45]. Furthermore, in line with previous studies on burnout consequences (e.g., [52]), teachers also reported experiencing negative emotions and physical and mental health complaints as the primary health outcomes, resulting in increased strain. Taken together, our qualitative findings align with international research and underscore the need for targeted structural interventions aimed at reducing workload, improving communication structures, and fostering a positive workplace environment grounded in trust, cooperation, and institutional support to mitigate occupational stress and prevent teacher burnout.

**Q3.**  *How Did the Social Environment of the Workplace Impact Teachers’ Occupational Health During the Pandemic?*

Our three-wave longitudinal analysis offers further empirical support for the evolving dynamics of burnout indicators and the protective function of a positive organizational climate. Consistent with the JD-R model [9], occupational stress was found to significantly explain the variance of emotional exhaustion, which in turn explained 28% of the variance of depersonalization, two of the core dimensions of burnout. However, personal accomplishment followed a distinct pattern: at T2, the variance in personal accomplishment was not significantly explained by either emotional exhaustion or depersonalization, but instead positively explained by organizational climate. Despite limitations related to cross-sectional data, these findings contribute to ongoing discussions in the burnout literature about the structural distinctiveness of personal accomplishment. Our results suggested that personal accomplishment may operate as a burnout dimension more responsive to job resources, particularly those of a relational and contextual nature [51].

Moreover, longitudinal moderation analyses partially supported our hypotheses, further sustaining these findings. Organizational climate moderated the relationship between occupational stress and teacher emotional exhaustion (confirming H1a), such that a more positive organizational climate was associated with a reduction in the adverse effects of work-related stress on emotional exhaustion. No significant moderation effects were found for depersonalization or personal accomplishment (rejecting H1b and H1c), but a main positive effect of organizational climate on personal accomplishment was confirmed.

This pattern reinforces theoretical assumptions from the Social Cognitive Theory [49] and the Structural Theory [52], which posit that burnout may emerge from a progressive erosion of professional efficacy and fulfillment at work. According to these frameworks, low personal accomplishment results as an initial reaction to distress when workers perceive a mismatch between professional demands and their individual or collective ability to meet them [50]. When the coping strategies employed are recurrently perceived as ineffective and feelings of low accomplishment at work intensify, emotional exhaustion may emerge, potentially evolving to depersonalization as a subsequent defensive coping mechanism, completing the three-dimensional syndrome of burnout [78].

These findings carry important implications. First, they underscore the need to prioritize the promotion of teachers’ professional efficacy as a central axis in occupational health promotion strategies to counteract burnout development [48]. Second, our results support the JD-R model’s focus on the dual role of job demands and resources. They show that enhancing job resources, such as organizational climate, can play a pivotal role in promoting personal accomplishment and buffering feelings of emotional exhaustion [40,62,79]. Finally, adding to prior literature, our findings provide empirical support for organizational-level interventions aimed at fostering a supportive, collaborative, and psychologically safe work environment [57,58,59,61]. In the current global context, marked by instability and unpredictability, the social environment of the workplace emerges as a crucial lever for protecting employee occupational health and well-being [37]. As such, policy and practice in educational settings should move beyond individual resilience frameworks and prioritize systemic organizational changes that reinforce supportive organizational climates as a buffer against chronic work-related stress and burnout.

## 5. Conclusions

### 5.1. Limitations

Our study is not without its limitations. It relied on a convenience, small, non-probabilistic, and geographically circumscribed sample drawn exclusively from public schools. These sample characteristics limit representativeness and generalizability, particularly with regard to the broader population of elementary school teachers (e.g., potential imbalances in baseline levels of stress and burnout). Although Portuguese educational regulations apply nationwide, teachers in different geographic, socioeconomic, and organizational contexts may have had different experiences that were not fully captured in the present study. Future research would benefit from larger, more diverse samples that include teachers from private schools and different regions. Probability-based or stratified sampling strategies would also enhance external validity. Using larger samples with a greater number of participating schools would also allow multilevel analytical approaches to be applied, explicitly accounting for the nested structure of teachers within schools and yielding more precise estimates of effects at the individual and organizational levels.

Data were collected exclusively online, using self-report questionnaires that required participants to recall past experiences. This may have introduced recall bias, social desirability effects, and common method variance. Furthermore, the use of repeated cross-sectional data on stress and burnout hinders the analysis of changes over time and limits causal interpretations. While these approaches facilitated data collection and a validation protocol was employed, future studies should use multi-method, fully paired, longitudinal data collection strategies, such as qualitative interviews, diary methods, or objective indicators. This would allow for the further exploration of developmental trajectories, reciprocal relationships, and temporal ordering of these variables and their associations, while reducing potential reporting biases.

Additionally, although different groups of work-related stressors were identified in the open-ended responses, teachers assessed their occupational stress on a general level in the quantitative measures. This limited the examination of the differential impact of specific stressor groups on burnout indicators. Future studies should address this limitation by incorporating more detailed assessments of stressors to enable a more nuanced analysis of how specific demands relate to teachers’ emotional exhaustion, depersonalization, and personal accomplishment. Furthermore, as our focus was on the moderating role of organizational climate as a job resource, specific job demands were not measured. This made it impossible to test the full JD–R model, including the buffering effects of job resources on specific job demands. This could be explored further in future studies.

Lastly, the absence of a baseline measure of organizational climate restricts conclusions regarding its potential buffering role in the stress–burnout relationship. Future research should include comprehensive baseline and follow-up assessments to allow for more robust testing of hypotheses. Despite these limitations, the present study provides valuable insights into teachers’ experiences and offers a solid empirical basis to inform future research by identifying key methodological priorities and substantive directions for advancing the study of occupational stress and burnout among elementary-school teachers.

### 5.2. Study Impact

Our study contributes to bridging a research gap by providing a multidimensional analysis of how Portuguese elementary school teachers navigated the early phases of the COVID-19 pandemic. While prior research has largely relied on cross-sectional or post hoc data, our inclusion of pre-pandemic baseline data (Fall 2019) alongside longitudinal and qualitative data offers a unique opportunity to explore the evolution of teachers’ occupational stress and burnout across two academic years of unprecedented disruption.

One of the critical findings of our study lies in the supporting evidence of perceived personal accomplishment as a pivotal dimension in the teacher burnout development process. Instead of simply being a result of emotional exhaustion and depersonalization, our data suggest that personal accomplishment may function as a distinct, resource-sensitive dimension of burnout [50,51], responsive to contextual factors such as organizational climate [80]. This result advances theoretical frameworks and can guide prompt action to foster teacher occupational health and well-being. Strengthening teachers’ professional efficacy can serve as an effective intervention to mitigate the risk of burnout, improve job performance, and retention [50]. Moreover, this finding should be considered in the context of teaching as a helping profession, where perceptions of competence, impact, and meaningful contribution might be closely tied to professional identity [45] and be valued job and personal resources [24,48]. In helping professions (e.g., healthcare, social work, teaching), personal accomplishment may occupy a more central position in the burnout process than in other occupations, where comparable experiences are typically conceptualized as professional efficacy, a slightly nuanced concept [81]. Although our study did not seek to make cross-occupational comparisons, this distinction highlights the relevance of future comparative research exploring whether the centrality of personal accomplishment is a profession-specific mechanism or represents a more generalizable pathway in burnout development. Addressing this question through comparative, multi-occupational designs would contribute to a more nuanced and integrative understanding of burnout across work contexts.

Additionally, our study revealed that social support and a positive work environment were critical job resources in mitigating the adverse effects of teacher work-related stress, acting against teacher burnout. The co-occurrence of insufficient social support with multiple job demands highlights the amplifying effect of resource deficits, suggesting that interventions must extend beyond individual resilience-building to encompass structural organizational changes [7]. The moderating role of organizational climate further sustains these conclusions. Thus, embedding social support within the organizational framework is critical to reducing burnout and improving educational outcomes. This suggests, aligning with prior literature (e.g., [57,58,59]), that interventions aimed at fostering a positive organizational climate, through positive leadership practices, colleagues’ and supervisors’ social support, and workplace policies aligned with teachers’ needs, should be prioritized.

Taken together, our results yield actionable recommendations for policymakers, school leaders, and practitioners. Interventions should be designed not only to address acute stressors brought on by the pandemic, but also to mitigate persistent, pre-existing structural challenges in the teaching profession that were heightened by this disruption. Drawing on the main results from both the qualitative and longitudinal data, we advocate for multi-tiered strategies including: investing in innovative approaches that leverage technology to optimize work processes and resource allocation to reduce workload and administrative burden; promoting collaborative, open, and transparent communication structures, and peer-mentorship and social integration initiatives to enhance collegial and leadership support; and developing institution-wide policies that support teacher autonomy and professional development to meet the new and unforeseen job demands that are arising from societal and work-related changes. These measures should be tailored to the specific challenges faced by teachers in different contexts, ensuring that interventions not only ease stressors but also cultivate sustainable and supportive work environments.

While data collection was completed in 2021, the significance of our findings remains unchanged. Our study captures a pivotal moment in teachers’ occupational health during the acute phase of the COVID-19 pandemic. However, the rapid changes in educational methods and demands caused by the pandemic have not only persisted but evolved. Hybrid teaching models, increasing digital integration, and heightened social, emotional, and behavioral challenges are now embedded features of many schooling systems, continuing to exert pressure on teachers. As recent global evidence sustains, teacher work-related stress remains high, driven by persistent structural changes in education and broader societal disruptions. Therefore, understanding how organizational climate can mitigate burnout remains imperative for designing effective support mechanisms in the post-pandemic BANI world.

In sum, our findings offer conceptual and practical insights that extend beyond the temporal bounds of the pandemic and provide valuable evidence to inform policies and professional development strategies aimed at fostering resilient educational environments and to plan for future challenges in education. Nonetheless, our data specifically pertain to teachers’ experiences between Fall 2019 and Summer 2021. Hence, any application to the current situation should be contextualized by the changes that have occurred since, rather than assumed as directly generalizable.

## Figures and Tables

**Table 1:** Means and standard deviations of teacher self-report of occupational stress and burnout indicators at the five data collection waves.

Outcome Variable	M (SD)				
	T1	T2	T3	T4	T5
Occupational stress	4.00 (0.86)	3.83 (1.02)	3.90 (0.99)	3.74 (0.96)	3.88 (1.01)
Burnout					
Emotional exhaustion	2.83 (1.06)	3.03 (1.30)	2.93 (1.26)	2.68 (1.23)	2.92 (1.21)
Depersonalization	0.71 (0.86)	0.60 (0.88)	0.62 (0.95)	0.56 (0.97)	0.66 (0.99)
Personal accomplishment	4.72 (0.67)	4.20 (0.90)	4.72 (0.69)	4.63 (0.80)	4.60 (0.79)
Organizational Climate	-	2.74 (0.37)	2.58 (0.30)	2.61 (0.30)	2.81 (0.32)

**Table 2:** Spearman correlation coefficients between occupational stress, burnout indicators, and organizational climate at T2 (*n* = 76).

	1.	2.	3.	4.	5.
Occupational stress	-				
Burnout					
Emotional exhaustion	0.54 **	-			
Depersonalization	0.02	0.28 *	-		
Personal accomplishment	0.02	−0.10	−0.26 *	-	
Organizational Climate	−0.04	−0.12	−0.09	0.38 **	-

* *p* < 0.05, ** *p* < 0.01.

## Data Availability

The datasets generated and analyzed during this study are available from the corresponding author upon reasonable request.

## Supplementary Materials

The following supporting information can be downloaded at: https://www.mdpi.com/article/10.3390/ijerph23010042/s1, Table S1: Category system based on the Job Demands and Resources Model [6], with an extended summary of quotations.

## Abbreviations

The following abbreviations are used in this manuscript:

BANI	Brittle, Anxious, Nonlinear, and Incomprehensible
JD-R	Job Demands and Resources
