# Supplementary material for: Addressing Teacher Occupational Health in Challenging Times: The Role of a Positive Organizational Climate in Buffering Teachers’ Burnout
Source: Int J Environ Res Public Health. 2025 Dec 28;23(1):42. doi: 10.3390/ijerph23010042 (PMC12841013; doi:10.3390/ijerph23010042)
Supplement: Supplementary file 1 [file ijerph-23-00042-s001.zip › ijerph-4010822-supplementary.pdf]

*Electronic Supplementary Material*

# Addressing Teacher Occupational Health in Challenging Times: The Role of a Positive Organizational Climate in Buffering Teachers' Burnout

Sofia Oliveira [1,*], Magda Sofia Roberto [2], Ana Margarida Veiga-Simão [2], and Alexandra Marques-Pinto [2].

[1] Business Research Unit (BRU), ISCTE—Instituto Universitário de Lisboa, 1649-026 Lisbon, Portugal
[2] CICPSI, Faculdade de Psicologia, Universidade de Lisboa, 1649-013 Lisbon, Portugal

* Correspondence: sofia.oliveira@iscte-iul.pt

**Table S1**

*Category system based on the Job Demands and Resources Model [6], with extended summary of quotations. Total recording units = 293.*

| **Categories (*f*; %)** | **Subcategories (*f*; %)** | ***% cases*** | ***Median (IQR)*** | **Examples of responses** |
|---|---|---|---|---|
| **Job Demands** (*f* = 257; 87.7%) | **Quantitative workload -** teachers experience of an excessive number of tasks, and administrative responsibilities within a limited timeframe, leading to a sense of urgency and perceived higher workload (*f* = 64; 24.9%) | 70.9% | 4.00 (1.00) | "Excessive workload"<br>"Loads of bureaucracy"<br>"Too many tasks at hand"<br>"Excessive demands and requests"<br>"Increased workload during working hours" |
| | **Demanding interactions with students -** teachers' difficulty in keeping close interactions with their students and manage | 45.5% | 4.00 (1.00) | "Low interaction with the students"<br>"Not being able to tutor students in person" |

| | | | | |
|---|---|---|---|---|
| | student behavior in distance learning. This includes lack of student engagement, absenteeism, the inability to interact with students in person and difficulties in providing support and maintain students' motivation and engagement (*f* = 34; 13.2%) | | | “Students missing classes”<br>“Keeping students focused online”<br>“Helping students in distance learning, especially those with more difficulties and fewer resources”<br>“Managing students’ behavior in online classroom” |
| | **Reorganization of work** – teachers experience changes in work structure, including curriculum modifications, administrative reorganizations, and adaptation to technology-driven teaching methods that disrupt their workflow (*f* = 30; 11.7%) | 43.6% | 4.00 (1.50) | “Lack of work routine”<br>“Having to work in an entirely different way than usual”<br>“Teaching remotely”<br>“Preparation of online classes” |
| | **Demanding interactions with parents -** teachers’ difficulty and strain in communicating with parents, particularly in cases of conflict, unrealistic expectations, and lack of cooperation or engagement in the educational process (*f* = 27; 10.5%) | 41.8% | 3.50 (1.00) | “Handling some parents”<br>“Lack of responsibility from some parents”<br>“Pressure from parents who constantly question me about everything”<br>“Utter lack of concern from some parents”<br>“Parents' presence in online classes” |
| | **Unfavorable work conditions -** presence of physical, environmental and technological constrains, such as inadequate workspaces, noise, excessive class sizes, inadequate technical support and lack of digital resources (e.g., computers, internet) (*f* = 24; 9.3%) | 25.5% | 4.00 (2.00) | “Absence of adequate IT equipment”<br>“Insufficient technological resources”<br>“No internet in schools”<br>“Internet and computer problems”<br>“Overcrowded classes”<br>“Insufficient educational support” |

| | | | |
|---|---|---|---|
| | | | “Poor facilities” |
| **IT constraints** – difficulties associated with the use of technology in teaching both by teachers and their students. It includes teacher strain related to the acquisition and use of digital competencies required for remote teaching (e.g., mastering specific online platforms or equipment), as well as students' lack of access to digital devices for effective participation in distance learning (*f* = 23; 8.9%) | 38.2% | 4.00 (2.00) | “Unfamiliarity with the new platforms”<br>“Having to learn how to work with new software”<br>“Students using their parents' mobile phones to do their work”<br>“Students with no access to computers or internet” |
| **Qualitative work overload -** increased complexity of cognitive and technical tasks that exceeds teachers’ competencies, preparation time, and/or available resources (*f* = 16; 6.2%) | 21.8% | 4.00 (1.00) | “Plan and prepare assignments, considering pedagogical differentiation”<br>“Managing pedagogical differentiation online”<br>“Finding engaging and stimulating activities for students” |
| **Work-life conflict –** teachers strain in balancing professional and personal and family life, in the context of remote work. It includes challenges in scheduling shared family resources, managing work time alongside household tasks, simultaneously handling teaching duties and family responsibilities, managing time effectively, and reconciling work demands with childcare (*f* = 13; 5.1%) | 16.4% | 4.00 (1.75) | “Balancing time spent on work with personal life”<br>“Simultaneously working as a teacher and running households”<br>“Managing work with childcare”<br>“Organizing how the various members of the family will use the same available resources” |
| **Unfavorable work schedule** – experience of irregular, excessive, and inflexible working hours that impact work-life balance and increase fatigue (*f* = 10; 3.9%) | 18.2% | 4.00 (1.75) | “Extended working hours”<br>“Not having a defined work schedule”<br>“Being available all the time” |

| | | | | |
|---|---|---|---|---|
| | **Emotional demands** – emotional demands associated with teaching, particularly in addressing students' needs and well-being. It includes excessive concern for students with learning difficulties who lack support at home, managing children's emotions, experiencing students' frustration, and worrying about their family situations (*f* = 7; 2.7%) | 12.7% | 4.00 (2.00) | "Being overly concerned about students with learning difficulties"<br>"Coping with children's emotions"<br>"Perceiving students' frustration"<br>"Increased social and emotional demands in the educational process" |
| | **Interpersonal conflicts** – teachers experience of interpersonal conflicts with their colleagues (*f* = 3; 1.2%) | 3.6% | 4.00 (2.00) | "Manage conflicts with colleagues"<br>"Managing tensions between students and their families" |
| | **Other job demands** - work-related stressors that include inadequate school and social policies that fail to support teachers effectively, excessive responsibility, accumulation of functions, and concerns about career stability (*f* = 6; 2.3%) | 10.9% | 5.00 (1.00) | "Accumulation of roles and responsibilities"<br>"Unnecessary but mandatory tasks"<br>"Devaluation of teachers"<br>"Career progression uncertainty" |
| **(Lack of) Job Resources** (*n* = 10; 3.4%) | **(Lack of) Social support –** teachers experience a lack of social support from colleagues and administrators, leading to feelings of detachment, isolation in the workplace, and reduced professional collaboration (*f* = 10; 100%) | 16.4% | 4.00 (0.50) | "Loss of contact with colleagues"<br>"Lack of interaction with colleagues"<br>"Demands from superiors"<br>"Isolation"<br>"Some colleagues' lack of support" |
| **Other social demands** (*f* = 5; 1.7%) - broader social adverse events that impact teachers' well-being. It includes references of being physically distant from family, stress and uncertainty related to the COVID-19 pandemic, and concerns about the health and safety of family members. | | 5.5% | 4.00 (1.50) | "Concern for family members"<br>"Being away from my family"<br>"The current national situation with COVID-19" |

| | | | | |
|---|---|---|---|---|
| **Outcomes (negative)** (*f* = 21; 7.2%) | **Negative emotions** - emotional distress resulting from work-related pressures, characterized by excessive anxiety, irritability, frustration, fear of not being able to meet students' needs, and uncertainty about the future (*f* = 9; 42.9%) | 10.9% | 2.00 (2.50) | "Frustration"<br>"Anxiety"<br>"Excessive irritability"<br>"Fear and uncertainty about what lie ahead" |
| | **Physical and Mental health complaints** - adverse health effects stemming from prolonged work strain, including physical exhaustion and pain, and mental fatigue (*f* = 8; 38.1%) | 12.7% | 4.00 (2.00) | "General body pain"<br>"Lower back pain"<br>"Mental exhaustion"<br>"Excessive tiredness" |
| | **Self-criticism** - heightened sense of personal inadequacy, excessive perfectionism, and self-imposed pressure to perform beyond one's capacity (*f* = 2; 9.5%) | 3.6% | 4.50 (0.50) | "Excessive perfectionism with myself"<br>"Not being able to do more and better" |
| | **Negative work-home interference** - insufficient time with family despite being physically present, causing emotional strain (*f* = 2; 9.5%) | 3.6% | 3.50 (0.50) | "Not spending time with my family"<br>"Not having time for my family even though I'm with them every day" |

*Note: f* = frequency of recording units coded in the (sub-)category; % cases = percentage of cases in which at least one recording unit was coded; IRQ = Inter-quartile Range
